# Identify Huntington’s disease associated genes based on restricted Boltzmann machine with RNA-seq data

**DOI:** 10.1186/s12859-017-1859-6

**Published:** 2017-10-11

**Authors:** Xue Jiang, Han Zhang, Feng Duan, Xiongwen Quan

**Affiliations:** 1 College of Computer and Control Engineering, Nankai University, Tongyan Road, Tianjin, 300350 China; 2 Tianjin Key Laboratory of Intelligent Robotics, Nankai University, Tongyan Road, Tianjin, 300350 China

**Keywords:** Restricted Boltzmann machine, Key genes associated to the disease progression, Huntington’s disease, RNA-seq data

## Abstract

**Background:**

Predicting disease-associated genes is helpful for understanding the molecular mechanisms during the disease progression. Since the pathological mechanisms of neurodegenerative diseases are very complex, traditional statistic-based methods are not suitable for identifying key genes related to the disease development. Recent studies have shown that the computational models with deep structure can learn automatically the features of biological data, which is useful for exploring the characteristics of gene expression during the disease progression.

**Results:**

In this paper, we propose a deep learning approach based on the restricted Boltzmann machine to analyze the RNA-seq data of Huntington’s disease, namely stacked restricted Boltzmann machine (SRBM). According to the SRBM, we also design a novel framework to screen the key genes during the Huntington’s disease development. In this work, we assume that the effects of regulatory factors can be captured by the hierarchical structure and narrow hidden layers of the SRBM. First, we select disease-associated factors with different time period datasets according to the differentially activated neurons in hidden layers. Then, we select disease-associated genes according to the changes of the gene energy in SRBM at different time periods.

**Conclusions:**

The experimental results demonstrate that SRBM can detect the important information for differential analysis of time series gene expression datasets. The identification accuracy of the disease-associated genes is improved to some extent using the novel framework. Moreover, the prediction precision of disease-associated genes for top ranking genes using SRBM is effectively improved compared with that of the state of the art methods.

**Electronic supplementary material:**

The online version of this article (doi:10.1186/s12859-017-1859-6) contains supplementary material, which is available to authorized users.

## Background

Neurodegenerative disease is a type of chronic degenerative disease in the central nervous system with the degenerative changes of the neuronal cells in brain and spinal cord. The symptoms of the neurodegenerative disease deteriorate slowly and eventually lead to death [1, 2]. Thereinto, the Huntington’s disease is due to a triplet (CAG) repeat elongation in the Huntington gene (IT15), which further affects numerous interactions between molecules. With the accumulation of the variant Htt protein in brain, a number of molecular pathways are affected in turn, resulting in neuronal malfunction and degeneration. Changes in Htt protein and the interactions between molecules are closely associated with the abnormalities of gene expression. It has been shown that there exist abnormalities of gene expression among the genes related to nerve conduction in the striatum tissue of Huntington’s disease individuals [3, 4]. Since the complexity of chronic disease, the molecular pathogenesis of Huntington’s disease is not entirely clear. Nevertheless, identifying the key genes associated with the disease deterioration can reveal useful insights into the disease pathogenesis.

The rapid development of high-throughput sequencing technologies, especially next-generation sequencing methods, provides possibility for us to explore the molecular mechanisms of complex disease on a genome-wide scale. However, because of the complex etiology of chronic diseases [5], the traditional disease-associated gene prediction methods cannot effectively identify the genes affected during the disease development. Generally, the disease-associated prediction methods roughly fall into three categories: network-based methods [6, 7], statistic-based methods [8–10], and machine learning methods [11, 12]. At present, as a branch of machine learning methods, the deep learning methods have become the most advanced technology in the field of computer vision, speech recognition and natural language processing. Deep learning methods use the hierarchical structure of deep neural network to conduct the nonlinear transfer of the input data, which could learn automatically the internal features that represent the original data [13, 14]. Compared with methods that are of manual designed features, the deep learning methods could improve the prediction accuracy. Recently, the deep learning methods have been introduced into the field of bioinformatics. Liang et al. [15] designed a multimodal deep belief network to conduct the integrative data analysis on multi-platform genomic data including gene expression data, miRNA expression data, and DNA methylation data. They used the model to detect a unified representation of latent features, capture both intra- and cross- modality correlations, and to identify key genes that may play distinct roles in the pathogenesis between different cancer subtypes. Cheng et al. [16] designed a miRNA prediction algorithm based on convolutional neural network (CNN). The CNN automatically extracts essential information from the input data while the exact miRNA target mechanisms are not well known. Experimental results demonstrated that the algorithm significantly improved the prediction accuracy.

During neurodegenerative disease development, gene expression level is affected by many factors, e.g. the environment, impaired metabolic pathways, protein mis-folding, etc [17–19]. Intuitively, identifying the key genes associated with the disease development is to screen the genes that are most seriously affected by these factors over with time. Consequently, the features that distinguish disease-related genes from non-disease-related genes could be represented by these factors. Extracting the deep hierarchical structure of the gene expression data and learning the important information represented by the decreased neurones in hidden layers are helpful to further understand the changes of gene expression during the disease development. In this paper, we designed a deep learning approach based on restricted Boltzmann machine to analyze the gene expression data [20], namely stacked restricted Boltzmann machine (SRBM). We used the unsupervised contrastive divergence algorithm (CD) to learn the parameters in each restricted Boltzmann machine [21, 22]. By maximizing the likelihood function, the probability distribution of the hidden layer variables fitted the probability distribution of the original data well. We trained the stacked restricted Boltzmann machine in a greedy layer-wise fashion [23]. Because the number of neurons in hidden layers is far smaller than that in the visible layer, we could reduce dimensions of the input data and capture useful high-level features of the input data at the same time. The gene expression level is manipulated by regulatory factors. In this work, we assume that the effects of regulatory factors can be captured by the hierarchical structure and narrow hidden layers of the SRBM. We used the model to rank the genes, aiming to make key genes that may play important roles in the pathogenesis of Huntington’s disease with high rankings. First, according to the differentially activated hidden neurons obtained by gene expression datasets at different time periods, we selected disease-associated factors. Then, we selected disease-associated genes according to the changes of the gene energy in SRBM at different time periods. Experimental results demonstrated that SRBM can detect the important information for differential analysis of time series gene expression datasets. The identification accuracy of the disease-associated genes is improved to some extent. Moreover, the prediction precision of disease-associated genes for top ranking genes using SRBM is effectively improved compared with that of the state of the art methods.

The presented study is organized as follows: The deep learning approach proposed in this paper is presented in “Methods” section. Experiments that analyze the performance of the stacked restricted Boltzmann machine and the overall discussion of the experimental results are reported in “Results and discussion” section. Conclusions are presented in “Conclusions” section.

## Methods

In this section, first, the stacked restricted Boltzmann machine model and the learning method are described. Next, we detailedly describe how the SRBM is used to extract the disease-associated genes with gene expression data at different disease stages. Finally, we present the parameter setting of the SRBM.

### Stacked restricted Boltzmann machine

#### Model

**Fig. 1:**
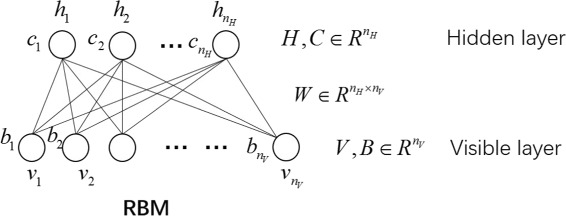
Schematic illustration of RBM

RBM is a kind of undirected probabilistic graphical model containing a layer of observable variables and a single layer of hidden variables [24]. In the RBM model (Fig. 1), each visible variable connects to every hidden variable, but no connections are allowed between any two variables within the same layer.

**Fig. 2:**
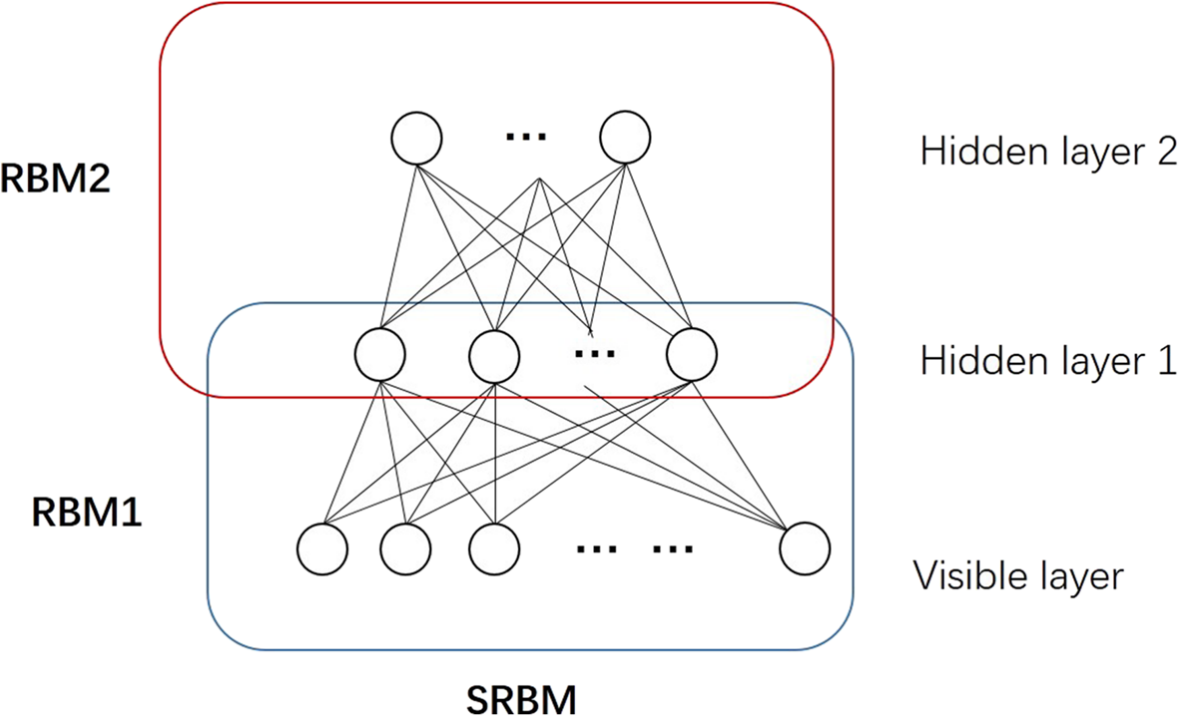
Schematic illustration of SRBM

In this study, we designed a stacked restricted Boltzmann machine to extract the hierarchical structures of gene expression dataset. The schematic illustration of SRBM is shown in Fig. 2. We add another RBM (denoted as RBM2 in Fig. 2) to the original RBM (denoted as RBM1 in Fig. 2). The input of visible layer in RBM2 is the output of hidden layer in RBM1. The dimension of gene expression data can be further reduced through the SRBM. As the gene expression data is real-valued data, we assume that the expression of genes obeys Gaussian distribution [15]. We use a Gaussian-Bernoulli RBM model for RBM1. However, variables in RBM2 are all binary numbers.

In the analysis of the gene expression dataset, the gene expression profile of a sample is *V*=(*v*_1_,*v*_2_,⋯,*v*_*n*__*V*_), where *v*_*i*_ represents the expression level of gene *i* and *n*_*V*_ is the number of genes. Here, *v*_*i*_ represents visible variable and *V* represents a layer of visible variables. *H*=(*h*_1_,*h*_2_,⋯,*h*_*n*__*H*_) denotes the layer of hidden variables, where *h*_*j*_ represents hidden variable and *n*_*H*_ is the number of hidden variables. The weight of the corresponding connection between hidden variable *h*_*j*_ and visible variable *v*_*i*_ is *w*_*ji*_. The weight matrix *W*=[*w*_*ji*_]_*n*__*H*_×*n*_*V*_ represents the parameter setting of weights between the hidden layer and the visible layer. Let *B*=(*b*_1_,*b*_2_,⋯,*b*_*n*__*V*_) be the bias vector of visible layer, where *b*_*i*_ stands for the bias of visible variable *v*_*i*_. Let *C*=(*c*_1_,*c*_2_,⋯,*c*_*n*__*H*_) be the bias vector of hidden layer, where *c*_*j*_ stands for the bias of hidden variable *h*_*j*_.

In RBM1 (Gaussian-Bernoulli RBM), the conditional distribution over the visible variables is usually supposed to be a Gaussian distribution whose mean is a function of the hidden variables [25, 26]. The conditional probability of a visible variable is

1 $$ p_{\theta}\left(v_{i}|H\right)= \mathcal{N}\left(\sum_{j=1}^{n_{H}}h_{j}w_{ji}+b_{i},\sigma_{i}^{2}\right),   $$

where *θ*=(*W*,*B*,*C*) represents the parameter setting of the model. Symbol *σ*_*i*_ is the standard deviation of Gaussian noise in visible variable *v*_*i*_.

The energy function of the RBM1 with binary hidden variables and real-valued visible variables can be defined as

2 $$ E_{\theta}(V,H)=\sum_{i=1}^{n_{V}}\frac{\left(v_{i}-b_{i}\right)^{2}}{2\sigma_{i}^{2}}-\sum_{j=1}^{n_{H}}c_{j} h_{j} -\sum_{i=1}^{n_{V}}\sum_{j=1}^{n_{H}}\frac{v_{i}}{\sigma^{2}_{i}}h_{j}w_{ji}.   $$

To simplify the parameter learning of the model, we standardized the input gene expression dataset, i.e., the average value of the visible variables *v*_*i*_ is equal to 0 and the variance of that is equal to 1 (*σ*_*i*_=1). In this way, the energy function in Eq. 2 can be rewritten as

3 $$ E_{\theta}(V,H)=\sum_{i=1}^{n_{V}}\frac{\left(v_{i}-b_{i}\right)^{2}}{2}-\sum_{j=1}^{n_{H}}c_{j} h_{j} -\sum_{i=1}^{n_{V}}\sum_{j=1}^{n_{H}}v_{i}h_{j}w_{ji}.  $$

The joint probability density function of (*V*,*H*) is given by

4 $$ p_{\theta}(V,H)= \frac{1}{Z(\theta)} e^{-E_{\theta}(V,H)},   $$

where *Z*(*θ*) is a normalizing constant known as the partition function, $Z(\theta)=\sum _{V,H}e^{-E_{\theta }(V,H)}$. It is important to state that the variables are under independent identical distribution. We need to get the conditional probability distribution of the visible variables due to the unobservability of the hidden layer, thus to solve the model. The edge probability distribution of the visible variables is given by

5 $$ p_{\theta}(V)=\sum_{H}p_{\theta}(V,H) = \frac{1}{Z(\theta)}\sum_{H}e^{-E_{\theta}(V,H)}.   $$

Since the gene expression data are very noisy, we discretized the gene expression values into binary values during the Gibbs sampling process. And we used binary activations instead of the real-valued visible units sampled from a Gaussian distribution which are usually seen as their activations. Because a binary activation contains less information than a real-valued gene expression, using the binary activation to represent a gene expression is helpful to distinguish the genes. This is a straightforward way to reduce noise in the gene expression data. The conditional probability density distributions can be easily obtained according to Eqs. 4 and 5. (The detail derivation process is given in Additional file 1).

6 $$ p\left(h_{k}=1|V\right) = \frac{1}{1 + e^{-\left(c_{k} + \sum_{i=1}^{n_{V}}w_{ki}v_{i}\right)}},   $$

7 $$ p(v_{k}=1|H) = \frac{1}{1 + e^{-\left(-0.5+b_{k} + \sum_{j=1}^{n_{H}}h_{j}w_{jk}\right)}}.   $$

In RBM2, *v*=(*v*_1_,*v*_2_,⋯,*v*_*n*__*v*_) represents the input layer (hidden layer 1 in Fig. 2) and *h*=(*h*_1_,*h*_2_,⋯,*h*_*n*__*h*_) denotes the output layer (hidden layer 2 in Fig. 2). The weight of the corresponding connection between output variable *h*_*j*_ and input variable *v*_*i*_ is *w*_*ji*_. The weight matrix *w*=[*w*_*ji*_]_*n*__*h*×*n*__*v*_ represents the parameter setting of weights between the output layer and the input layer. Let *b*=(*b*_1_,*b*_2_,⋯,*b*_*n*__*v*_) be the bias vector of input layer, where *b*_*i*_ stands for the bias of variable *v*_*i*_. Let *c*=(*c*_1_,*c*_2_,⋯,*c*_*n*__*h*_) be the bias vector of output layer, where *c*_*j*_ stands for the bias output variable *h*_*j*_.

As the variables in RBM2 are all binary, the energy function of the RBM2 model is defined as

8 $$ E_{\theta}(v,h)=-\sum_{i=1}^{n_{v}}b_{i} v_{i}-\sum_{j=1}^{n_{h}}c_{j} h_{j} -\sum_{i=1}^{n_{v}}\sum_{j=1}^{n_{h}}h_{j}w_{ji} v_{i}.   $$

In the same way, we get the following conditional probability density distributions

9 $$ p\left(h_{k}=1|v\right) = \frac{1}{1 + e^{-\left(c_{k} + \sum_{i=1}^{n_{v}}w_{ki}v_{i}\right)}},  $$

10 $$ p\left(v_{k}=1|h\right) = \frac{1}{1 + e^{-\left(b_{k} + \sum_{j=1}^{n_{h}}h_{j}w_{jk}\right)}}.  $$

#### Learning

Training the RBM model means to learn the parameters of the model, making sure that the probability density distribution of the hidden variables fit that of the variables in the visible layer well. Physically, the energy function of the system is minimized when the system reaches a steady state. Mathematically, the goal of RBM training is to maximize the logarithmic likelihood function. For such a type of optimization problem, we use gradient up method to learn the parameters of the model.

11 $$ \theta := \theta +\eta \frac{\partial log p_{\theta}(V)}{\partial \theta},   $$

12 $$ \frac{\partial log p_{\theta}(V)}{\partial \theta} \,=\, -\!\left\langle \frac{\partial E_{\theta}(V,H)}{\partial \theta} \right\rangle_{p_{\theta}(H|V)} \!+ \left\langle \frac{\partial E_{\theta}(V,H)}{\partial \theta} \right\rangle_{p_{\theta}(V,H)},   $$

where *η* is learning rate, $\left \langle \frac {\partial E_{\theta }(V,H)}{\partial \theta } \right \rangle _{p_{\theta }(H|V)}$ is the expectation of energy gradient function $\frac {\partial E_{\theta }(V,H)}{\partial \theta }$ under the condition distribution *p*_*θ*_(*H*|*V*), and $\left \langle \frac {\partial E_{\theta }(V,H)}{\partial \theta } \right \rangle _{p_{\theta }(V,H)}$ is the expectation of energy gradient function under the joint distribution *p*_*θ*_(*V*,*H*). Since the hidden variables cannot be directly observed, we use CD-*k* algorithm to approximately estimate the probability *p*_*θ*_(*V*) though Gibbs sampling in *k* steps [21, 22], thus to obtain the solution of $\left \langle \frac {\partial E_{\theta }(V,H)}{\partial \theta } \right \rangle _{p_{\theta }(V,H)}$. For sample *V*, the initial values of visible layer is *V* ^(0)^=*V*. We use *V* ^(*k*)^ to denote the sample obtained by CD-*k*.

The gradients for sample *V* in one iterative process are given by (The detail derivation process is given in Additional file 1).

13 $$ {} \frac{\partial log p_{\theta}(V)}{\partial w_{ij}} = p\left(h_{i}=1|V^{(0)}\right)v_{j}^{(0)}-p\left(h_{i}=1|V^{(k)}\right)v_{j}^{(k)},   $$

14 $$ \frac{\partial log p_{\theta}(V)}{\partial b_{i}} = v_{i}^{(0)}-v_{i}^{(k)},   $$

15 $$ \frac{\partial log p_{\theta}(V)}{\partial c_{i}} = p\left(h_{i}=1|V^{(0)}\right)-p\left(h_{i}=1|V^{(k)}\right).   $$

In this study, we use mini-batch strategy to learn parameters in the RBM. We use sample set *S*={*V* ^1^,*V* ^2^,⋯,*V* ^*n*^} to train the model one batch. Here *n*_*block*_=*n* represents the size of mini-batch. The gradient calculation formula for one iteration is shown below

16 $$ \frac{\partial log L_{s}}{\partial \theta} = \sum_{t=1}^{n} \frac{\partial\left(log p(V^{t})\right)}{\partial \theta},   $$

where *L*_*s*_=*p*_*θ*_(*S*) is the likelihood function of product edge probability density distributions, *V* ^*t*^ represents the *t*-th sample. The gradients for *S* in one iteration are given by

17 $$ {\begin{aligned} \frac{\partial log L_{s}}{\partial w_{ij}} = \sum_{t=1}^{n}\left[p\left(h_{i}=1|V^{t(0)}v_{j}^{t(0)}-p\left(h_{i}=1|V^{t(k)}\right)v_{j}^{t(k)}\right.\right], \end{aligned}}  $$

18 $$ \frac{\partial log L_{s}}{\partial b_{i}} = \sum_{t=1}^{n}\left[ v_{i}^{t(0)}-v_{i}^{t(k)}\right],   $$

19 $$ \frac{\partial log L_{s}}{\partial c_{i}} = p\left(h_{i}=1|V^{t(0)}\right)-p\left(h_{i}=1|V^{t(k)}\right).   $$

In summary, the detail training process of the RBM is shown below.



We trained the stacked restricted Boltzmann machine in a greedy layer-wise fashion [23]. We first trained the RBM1 according to the above training process (see Algorithm 1), then trained RBM2 in the same way.

### Identification of key genes

In our study, the regulatory factors are seen as high-level features which could be captured by the hierarchical structure and narrow hidden layers of the SRBM. On the one hand, the differentially activated hidden neurons are important for distinguishing different disease stage samples. On the other hand, the neurons differential activation indicates that the regulatory factors change greatly during the disease development. So, we select disease-related regulatory factors according to the differentially activated neurons in the hidden layers.

Biologically, the connections among neurons in one functional neural circuit are more strong. In fact, it has also been shown that the high-level hidden units in RBM tend to have strong positive weights to similar features in the visible layer [27]. In an SRBM model, the connections from a visible unit in the input layer to the high-level features (disease-related regulator factors) are seen as the connections in a functional neural circuit. And we use the energy of the neural circuit in the SRBM to measure the property of the input unit (represent a gene). Since the hidden units were activated very differently along with the disease progression, the energy of the neural circuit changed greatly. It suggests that the gene expression has been greatly affected during the disease development. Based on the above analysis, we rank the genes according to the energy changes at different time periods. The higher the ranking of gene it is, the more likely the disease-related gene it is.

Let $x_{i}^{s}$ denote the activated frequency of neuron *i* in the first hidden layer, using the gene expression data of *s* time period samples. Symbol $y_{j}^{s}$ denotes the activated frequency of neuron *j* in the second hidden layer, i.e., the output layer. Let $E_{g}^{s}$ denote the energy of gene *g* at *s* time period. According to Eqs. 3 and 8, the energy of gene *g* is given by

20 $$ {{} \begin{aligned} E_{g} = \frac{\left(v_{g}-b_{1,g}\right)^{2}}{2} - \sum_{j=1}^{n_{H}}h_{1,j}w_{1,jg}v_{g}&-\sum_{i=1}^{n_{v}}b_{2,i} v_{2,i} \\&-\sum_{i=1}^{n_{v}}\sum_{j=1}^{n_{h}}h_{2,j} w_{2,ji} v_{2,i},  \end{aligned}}  $$

where *b*_1,*i*_, *h*_1,*i*_, *w*_1,*ji*_ represent the parameters in RBM1 and *b*_2,*i*_, *v*_2,*i*_, *h*_2,*i*_, *w*_2,*ji*_ represent the parameters in RBM2. Since the energy caused by the bias of the hidden layer in RBM1 is same for all genes, we omit the term in the calculation formula of gene energy.

The energy change of gene *g* at different time periods is computed by

21 $$ C_{g} = \left|\frac{1}{|s_{1}|}\sum_{i=1}^{s_{1}} E^{s_{1}}_{g} - \frac{1}{|s_{2}|}\sum_{i=1}^{s_{2}} E^{s_{2}}_{g}\right|,   $$

where *s*_*i*_ denotes the samples at *i* time period. The details for identifying key genes are shown below: **Step 1.** Rank the two hidden layer neurons in descending order according to the difference of the activated frequency between different time periods, respectively. We select the top ranked neurons in the ranked lists as the differentially activated neurons, respectively. The neurons that are not differentially activated in the two hidden layers are all set to 0 in any case. **Step 2.** Compute the energy changes of gene *g* at different time periods according to Eq. 21. Rank genes in descending order according to the energy changes of genes.

### Parameter setting

Here, we initialize parameters in SRBM according to empirical studies in deep learning literature. The initialization weights obey Gaussian distribution *N*(0,0.01). The initialization bias variables are set to 0. The learning rate *η*=0.5. The number of hidden neurons is usually about one tenth of visible neurons. In this study, the number of variables in the first hidden layer is 400 and that of the second hidden layer is 20. Moreover, the number of sampling steps in CD-*k* is set to be *k*=1.

## Results and discussion

We used the SRBM to analyze the gene expression data of Huntington’s disease mice at different time periods. In this section, first, we briefly introduce the dataset used in this study. Second, we demonstrate the experimental results using SRBM. Then, we compare the performance of SRBM with other computational methods. Finally, we analyze and discuss the results of SRBM in detail.

### Gene expression data

The gene expression dataset used in this study were downloaded from http://www.hdinhd.org, which were obtained from the striatum tissue of Huntington’s disease mice by using RNA-seq technology. The genotype of Huntington’s disease mice is ployQ 111. There are 8 samples of 2-month-old mice and 8 samples of 6-month-old Huntington’s disease mice. We conducted a preprocessing step to filter out noisy and redundant genes by selecting the genes with large mean value and variance of the 16 samples. Finally 4433 genes from the total 23,351 genes were left for further analysis. The data of modifier genes were from [28], which contained 520 genes, including 89 disease-related genes and 431 non-disease-related genes.

### The results of SRBM

**Fig. 3:**
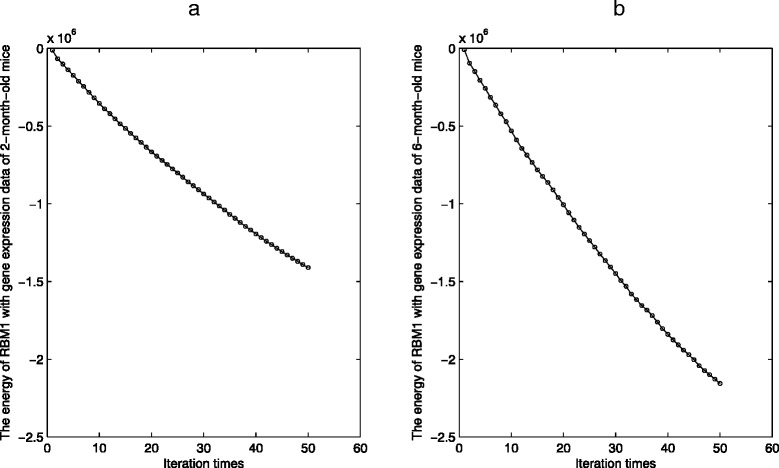
The energy change of RBM1. **a** The energy change of RBM1 with gene expression data of 2-month-old Huntington’s disease mice. **b** The energy change of RBM1 with gene expression data of 6-month-old Huntington’s disease

**Fig. 4:**
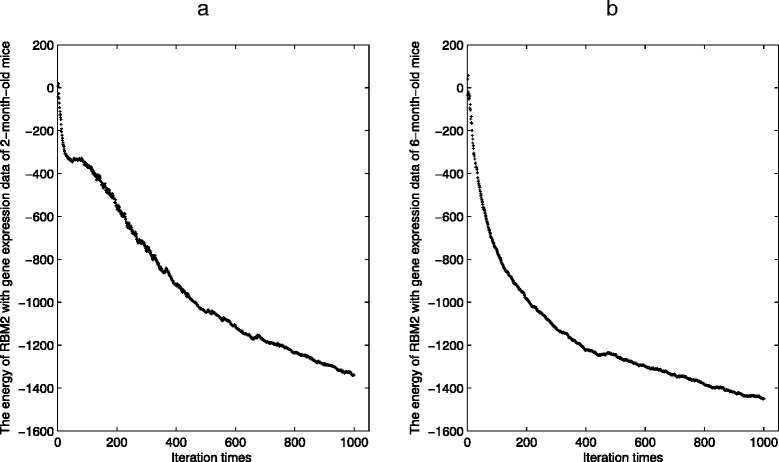
The energy change of RBM2. **a** The energy change of RBM2 with gene expression data of 2-month-old Huntington’s disease mice. **b** The energy change of RBM2 with gene expression data of 6-month-old Huntington’s disease

Figures 3 and 4 show the energy changes of RBM1 and RBM2 along with every iteration during the parameter training process. From Figs. 3 and 4, we can see that the changes become small with the increasing of iterations. In this study, since there are large amounts of parameters in RBM1, the iteration times of RBM1 are preset to be 50 to reduce computational time and avoid over-fit. The iteration times of RBM2 are preset to be 400 to avoid over-fit.

We statisticed the differentially activated frequency of neurons in the hidden layers using SRBM with gene expression datasets at different time periods. The results are shown in Table 1. Compared with the differentially activated frequency of neurons in the hidden layer 1, that in the hidden layer 2 is much larger. The number of neurons, whose differentially activated frequency in hidden layer 1 is 3, is too small to be used to distinguish samples at different time periods. It is better to use the neurons with largest differentially activated frequency in the hidden layer 2 to distinguish samples at different time periods, thus to identify the key genes that may be seriously affected during the disease progression.

**Table 1:** The number of neurons that are of the same differentially activated frequency using SRBM with different time period samples

Differentially activated frequency	Hidden layer 1	Hidden layer 2
5	0	5
4	0	2
3	4	3
2	57	3
1	199	4
0	140	3

**Fig. 5:**
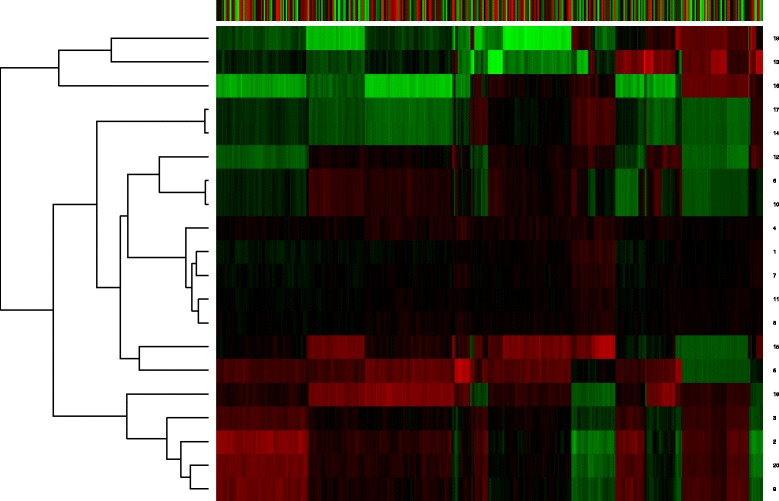
Heatmap of weight matrix of RBM2 with 2-month-old gene expression data. The weight matrix is obtained using SRBM with gene expression data of 2-month-old Huntington’s disease mice

**Fig. 6:**
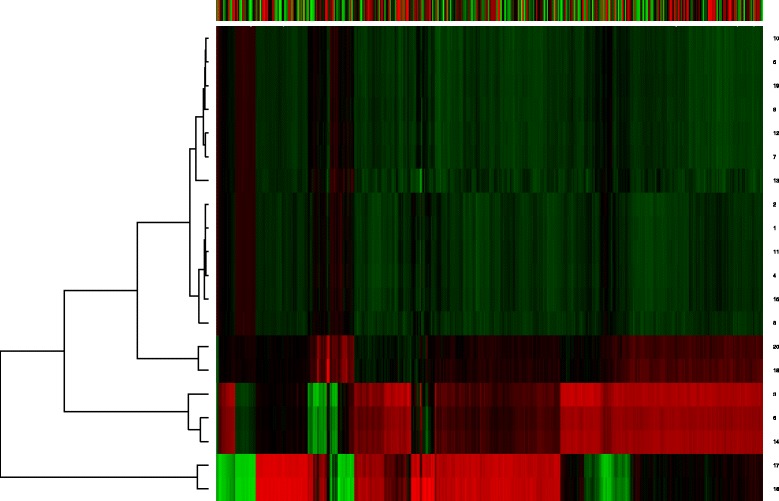
Heatmap of weight matrix of RBM2 with 6-month-old gene expression data. The weight matrix is obtained using SRBM with gene expression data of 6-month-old Huntington’s disease mice

Furthermore, we draw heatmaps of the weight matrices of RBM2 to investigate the deep structure difference between the gene expression data of Huntington’s disease mice at different time periods. The weight matrices are obtained by using SRBM with gene expression datasets of Huntington’s disease mice at different time periods (Figs. 5 and 6). The numbers in the left of the heatmap represent the corresponding neuron in the output layer. From Figs. 5 and 6, we can clearly see that there are significant difference between the two heatmaps. It suggests that the gene expression changes complicatedly during the disease progression.

### Performance comparison between SRBM with other methods

To verify the performance of SRBM, we conducted other experiments using the original RBM method, t-test method [10], fold change rank-product method (FC-RP) [10], and joint non-negative matrix factorization meta-analysis method (jNMFMA) [11] with the gene expression data. We use true positive rate (TPR), false positive rate (FPR), precision, and recall to evaluate the prediction accuracy of disease-associated genes. TPR is defined as the ratio of correctly predicted disease genes to all disease genes. FPR is defined as the ratio of incorrectly predicted disease genes to all non-disease genes. Precision is defined as the ratio of correctly predicted disease genes to all the predicted disease genes. Recall is defined as the ratio of correctly predicted disease genes to all disease genes. The receiver operating characteristic (ROC) curves were created by plotting TPR versus FPR. The precision-recall (PR) curves were created by plotting precision versus recall. The area under the ROC curve (AUC) and the area under the precision-recall curve (AUPR) were used as measures of the prediction accuracy [29].

To test the reasonability of the assumption in this study, we used all neurons in hidden layers to compute the gene energy while overlooking one third weak connections that from one neuron to all the neurons of the next layer. The corresponding experiments are denoted as SRBM-I. On the other hand, we selected differentially activated neurons at different time periods as factors that manipulate the expression of all genes during the disease progression, 61 neurons were selected in the first hidden layer with differentially activated frequency larger than 1, and 5 neurons were selected in the second hidden layer with differentially activated frequency larger than 5. Then, we computed the energy for each gene. The corresponding experiments are denoted as SRBM-II. Note that we use RBM-I and RBM-II to denote the experiments using the original RBM model.

**Fig. 7:**
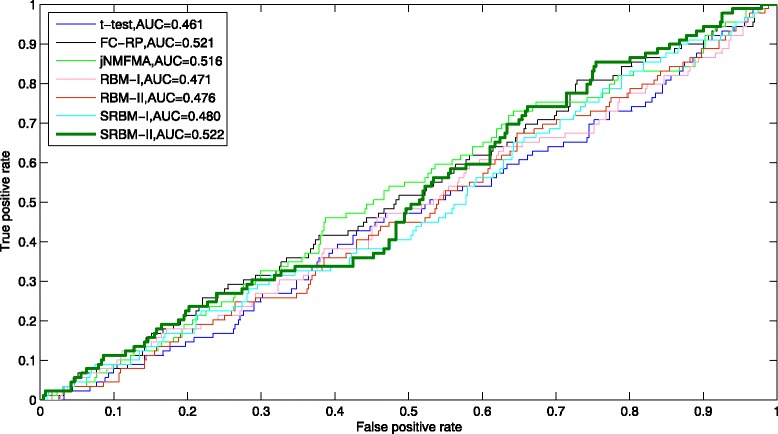
ROC curves. The ROC curves of the prediction results using t-test, FC-RP, jNMFMA, RBM-I, RBM-II, SRBM-I and SRBM-II

From Fig. 7, we can see that the ROC cures of the seven methods are similar. The AUCs of these methods are around 0.5. It illustrates that these methods cannot separate the disease genes from non-disease genes in the modifier gene set. It also indicates that the expression of genes change complicatedly during the disease development. Nevertheless, the AUC of SRBM-II is mildly improved compared with that of the other six methods.

**Fig. 8:**
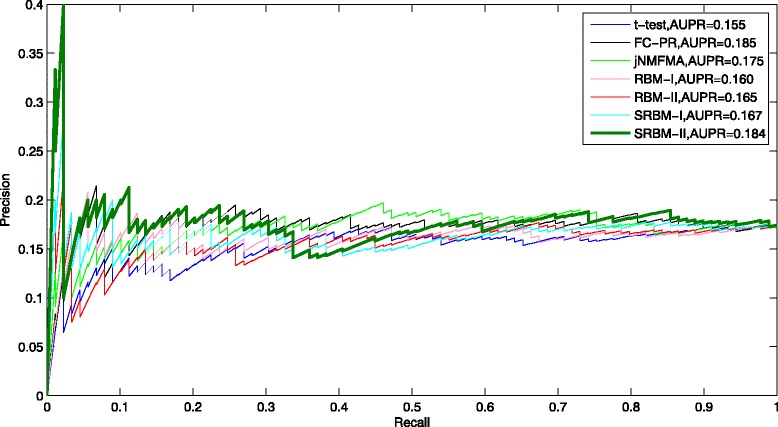
Rank-product curves. The RP curves of the prediction results using t-test, FC-RP, jNMFMA, RBM-I, RBM-II, SRBM-I and SRBM-II

From Fig. 8, the PR curves of the seven methods are similar to some extent. However, the prediction precision for top ranked genes of the seven methods are clearly distinct. The prediction precision of SRBM-II is significantly higher for top ranked genes compared with that of the other six methods.

**Fig. 9:**
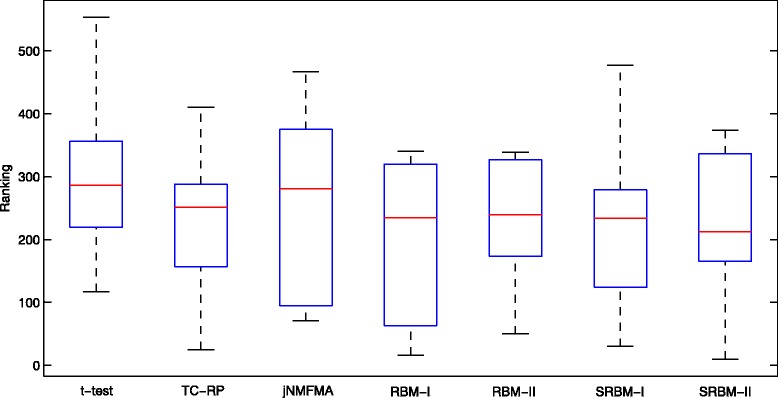
Boxplots of the rankings of top ranked 10 disease genes. The rankings are obtained using different methods, including t-test, FC-RP, jNMFMA, RBM-I, RBM-II, SRBM-I and SRBM-II

We further investigate the distributions of the rankings of top ranked 10 disease genes in the ranked lists obtained by using the seven methods, respectively (Fig. 9). From Fig. 9, we can roughly know the rankings of the top ranked disease genes. Although the distributions obtained by these methods are similar, SRBM-II makes the disease genes get mild higher rankings compared with the other six methods.

In total, the performance of SRBM-II is moderately better than other methods. From Figs. 7, 8 and 9, we can know that the performance of SRBM-II is better than SRBM-I. It suggests that we improved the prediction accuracy by selecting the differentially activated neurons, which are assumed to be disease-associated factors in our study. We can also know that the performance of SRBM methods are better than RBM methods. It verifies that we effectively separated some noisy factors from the gene expression dataset, using the deep structure of SRBM.

We also statisticed the overlapped degree of top ranked 500 genes between any two ranked lists, the results are shown in Table 2. It can be clearly seen that the overlapped degrees between any two ranked lists (except for that between SRBM-I and SRBM-II) are all small. However, the overlap degrees between jNMFMA and SRBM methods are smaller than that between others. The jNMFMA assumes that the gene expression is a weighted linear combination of metagenes. The jNMFMA selects disease-associated genes through differentially regulated metagenes. SRBM selects disease-associated genes according to the energy changes at different disease states. Since the basic assumptions of the two models are greatly different, the overlapped degrees of top ranked genes between the two ranked lists are smaller.

**Table 2:** The number of overlapped genes (the degree of overlap) of top ranked 500 genes between any two ranked lists obtained using t-test, FC-RP, jNMFMA, RBM-I, RBM-II, SRBM-I, and SRBM-II

	FC-RP	jNMFMA	RBM-I	RBM-II	SRBM-I	SRBM-II
t-test	81 (16.2%)	36 (7.2%)	73 (14.6%)	74 (14.8%)	75 (15%)	73 (14.6%)
FC-RP		114 (22.4%)	28 (5.6%)	22 (4.4%)	38 (7.6%)	40 (8.0%)
jNMFMA			6 (1.2%)	8 (1.6%)	5 (1.0%)	9 (1.8%)
RBM-I				344 (68.8%)	252 (50.4%)	214 (42.8%)
RBM-II					245 (49.0%)	248 (49.6%)
SRBM-I						351 (70.2%)

The top ranked 500 genes in different ranked lists share 4 common genes: Chmp1b, Poldip3, Lrrtm1 and Slc44a1. According to the annotation of Gene Ontology, the molecule function of Chmp1b is protein domain specific binding, that of Lrrtm1 is protein kinase inhibitor activity, that of Poldip3 is nudeotide binding, and that of Slc44a1 is choline transmembrane transporter activity. The functions of the four genes are all related to protein transportation. Those genes may be related to the disturbance of intracellular protein trafficking in Huntington’s disease individuals [30].

### Enrichment analysis

**Fig. 10:**
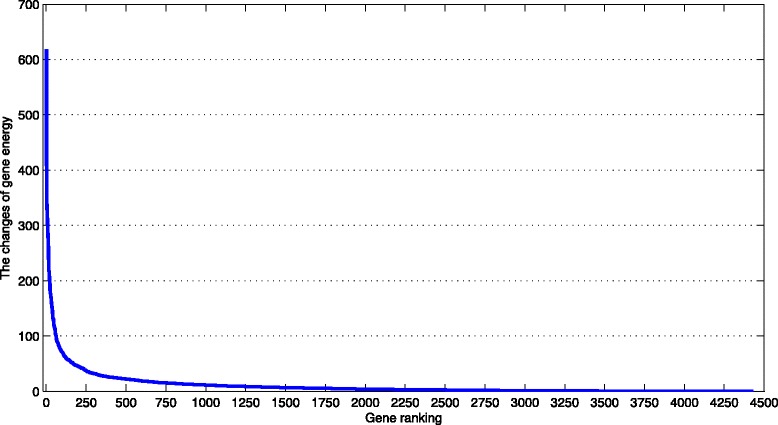
The changes of gene energy. The gene ranking is obtained by using SRBM-II based on the changes of gene energy at different time periods

According to Fig. 10, it is obvious that the changes of gene energy for the top ranked 100 genes are significantly larger. Combined with Fig. 8, we known that the higher the ranking of gene it is, the more precise the prediction accuracy of disease-related gene it is. To avoid introducing too many false positives, we chose the top ranked 100 genes in the ranked list obtained by using SRBM-II to conduct enrichment analysis. We used the functional annotation clustering tool through DAVID [31] to annotate the functions of those genes, the result can be seen in Table 3. The annotations listed in the table are cellular component from GOTERM. From Table 3, we can see that those genes are related to membrane, synapse and cell junction. It suggests that the cellular form changes greatly during the Huntington’s disease progression and deterioration. In fact, the connections between neurons get sparse, and the neurons finally died during the Huntington’s disease deterioration [32, 33].

**Table 3:** The functional annotation clustering of the top ranked 100 genes in the ranked list obtained using SRBM-II

Annotation	Annotation	Genes-	*P*-value	Benjamini
cluster		included		
Annotation	Membrane	60	7.2E-8	7.3E-6
cluster 1	Plasma membrance	42	5.0E-5	1.1E-3
Annotation	Synapse	14	8.2E-7	3.3E-5
cluster 2	Postsynaptic density	10	2.2E-6	7.3E-5
	Dendritic spine	7	7.8E-5	1.6E-3
	Cell junction	11	2.3E-3	2.5E-2
	Synaptic vesicle	4	2.3E-2	1.8E-1
	Postsynaptic membrane	4	9.0E-2	4.0E-1
Annotation	Cell-cell adherens junction	8	8.0E-4	1.3E-2
cluster 3				

## Conclusions

In this paper, we designed a stacked restricted Boltzmann machine to detect the hierarchical structures and to capture the important information for differential analyzing gene expression datasets of Huntington’s disease mice at different time periods. We also proposed a new framework to identify the key genes that may be affected by the disease progression. Experimental results verify the feasibility of the assumption in this study. It also demonstrates that the performance of SRBM-II is mildly better than other traditional methods. Besides the exploratory analysis of the disease molecular mechanisms through enrichment analysis, we also conducted a integrated analysis on the ranked lists obtained by the seven methods. We found that four genes (Chmp1b, Poldip3, Lrrtm1 and Slc44a1) related to protein transportation are seriously affected during the disease progression.

## Additional file

Additional file 1 Supplementary Material. The detail derivation process for solving the gradients of RBMs learning is given in the Supplementary Material. (PDF 321 kb)

## Abbreviations

AUC: The area under the ROC curve
AUPR: The area under the PR curve
CD: Contrastive divergence
CNN: Convolutional neural network
FC-RP: Fold change rank-product
FPR: False positive rate
jNMFMA: Joint non-negative matrix factorization neta-analysis method
PR: Precision-recall
RBM: Restricted Boltzmann machine
ROC: Receiver operating characteristic
SRBM: Stacked restricted Boltzmann machine
TPR: Stacked restricted Boltzmann machine
