# Supplementary material for: Identify Huntington’s disease associated genes based on restricted Boltzmann machine with RNA-seq data
Source: BMC Bioinformatics. 2017 Oct 11;18:447. doi: 10.1186/s12859-017-1859-6 (PMC5637347; doi:10.1186/s12859-017-1859-6)
Supplement: Supplementary file 1 — Supplementary Material. The detail derivation process for solving the gradients of RBMs learning is given in the Supplementary Material. (PDF 321 kb) [file 12859_2017_1859_MOESM1_ESM.pdf]

Supplementary material for "Identify Huntington's disease associated genes based on restricted Boltzmann machine with RNA-seq data"

Xue Jiang[1,2], Han Zhang[1,2], Feng Duan[1,2], Xiongwen Quan[1,2*]

[1] College of Computer and Control Engineering, Nankai University, Tianjin, 300350, China

[2] Tianjin Key Laboratory of Intelligent Robotics, Nankai University, Tianjin, 300350, China

[*] Correspondence: quanxw@nankai.edu.cn

1 Restricted Boltzmann machine

Restricted Boltzmann machines (RBMs) are undirected probabilistic graphical models containing a layer of observable variables and a single layer of hidden variables [1]. It allows no connections between any visible variables or between any hidden variables. Fig. S1 shows the graph structure of RBMs. The RBM is one kind of energy-based models.

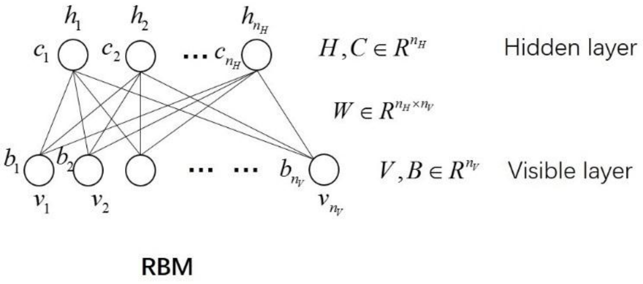

Figure S1. Schematic illustration of an RBM.

1.1 RBMs for binary variables

We firstly begin with the binary version of the RBMs. The energy function for the RBM is given by

$$\begin{aligned} E_\theta(v,h) &= -\sum_{i=1}^{n_v} b_i v_i - \sum_{j=1}^{n_h} c_j h_j - \sum_{i=1}^{n_v}\sum_{j=1}^{n_h} h_j w_{ji} v_i \\ &= -b^T v - c^T h - h^T W v \end{aligned}, \quad (1)$$

where $v=\left(v_1,v_2,\cdots,v_{n_v}\right)^T$ represents the layer of visible variables, and $h=\left(h_1,h_2,\cdots,h_{n_h}\right)^T$ represents the layer of hidden variables. The weight of the corresponding connection between hidden variable h_j and visible variable v_i is w_{ji}. The weight matrix $W=\left[w_{ji}\right]_{n_h\times n_v}$ represents the parameter setting of weights between the hidden layer and the visible layer. The vector $b=\left(b_1,b_2,\cdots,b_{n_v}\right)^T$ is the bias vector of visible layer, where b_i stands for the bias of visible variable v_i. And the vector $c=\left(c_1,c_2,\cdots,c_{n_h}\right)^T$ is the bias vector of visible layer, where c_j stands for the bias of hidden variable h_j. $\theta=(W,b,c)$ represents the parameter setting of the model.

The joint probability distribution using the energy function is given by

$$p_\theta\left(v,h\right)=\frac{e^{-E_\theta\left(v,h\right)}}{Z\left(\theta\right)}, \quad (2)$$

where $Z\left(\theta\right)$ is the partition function that ensures $\sum_{v,h}p_\theta(v,h)=1$, and $Z\left(\theta\right)=\sum_{v,h}e^{-E_\theta\left(v,h\right)}$. It is important to state that the variables are under independent and identically distributions. We need to get the conditional probability distribution of the visible variables due to the unobservability of the hidden layer, thus to solve the model.

The edge probability distribution of the visible variables is given by

$$p_\theta\left(v\right)=\sum_h p_\theta\left(v,h\right)=\frac{1}{Z(\theta)}\sum_h e^{-E_\theta\left(v,h\right)} \quad . \quad (3)$$

Training a RBM model means to learn the parameters of the model, and make the probability density distribution of the hidden variables better fit that of the variables in the visible layer. In other words, the probability density distribution of the hidden variables obtained through the RBM model should be consistent with that represented by the training samples as much as possible. Physically, the energy function of the system is minimized when the system reaches a steady state. Mathematically, the training goal for RBM is to maximize the logarithmic likelihood function.

The likelihood function by averaging all training samples is shown below

$$L_{\theta,S}=\prod_{t=1}^{T}p_\theta\left(v^t\right), \quad (5)$$

where v^t denotes the t-th sample, $t=1,2,\cdots,T$.

Because of the log-likelihood function has the same monotonicity as the likelihood function, we use log-likelihood function to simplify the calculation procedure. The log-

likelihood function is shown below:

$$\begin{aligned}\theta^* &= \arg\max_{\theta} \ \log L_S(\theta) \\ &= \arg\max_{\theta} \ \sum_{t=1}^{T} \log p_\theta(v^t)\end{aligned} . \quad (6)$$

We use gradient up method to learn the parameters,

$$\theta = \theta + \eta \frac{\partial(\log L_S(\theta))}{\partial\theta}, \quad (7)$$

where η is learning rate.

We first compute the gradient of one sample. The log-likelihood function is given by

$$\begin{aligned}L(\theta) &= \log p_\theta(v) \\ &= \log\left(\frac{1}{Z(\theta)}\sum_h e^{-E_\theta(v,h)}\right) \\ &= \log\left(\sum_h e^{-E_\theta(v,h)}\right) - \log(Z(\theta)) \\ &= \log\sum_h e^{-E_\theta(v,h)} - \log\sum_{v,h} e^{-E_\theta(v,h)}\end{aligned} . \quad (8)$$

So,

$$\begin{aligned}\frac{\partial L(\theta)}{\partial\theta} &= \frac{\partial}{\partial\theta}\left(\log\sum_h e^{-E_\theta(v,h)}\right) - \frac{\partial}{\partial\theta}\left(\log\sum_{v,h} e^{-E_\theta(v,h)}\right) \\ &= -\sum_h \frac{e^{-E_\theta(v,h)}}{\sum_h e^{-E_\theta(v,h)}} \frac{\partial E_\theta(v,h)}{\partial\theta} + \sum_{v,h} \frac{e^{-E_\theta(v,h)}}{\sum_{v,h} e^{-E_\theta(v,h)}} \frac{\partial E_\theta(v,h)}{\partial\theta} \\ &= -\sum_h p_\theta(h|v)\frac{\partial E_\theta(v,h)}{\partial\theta} + \sum_{v,h} p_\theta(v,h)\frac{\partial E_\theta(v,h)}{\partial\theta}\end{aligned} . \quad (9)$$

where $\sum_h p_\theta(h|v)\frac{\partial E_\theta(v,h)}{\partial\theta}$ is the expectation of the energy gradient function of $\frac{\partial E_\theta(v,h)}{\partial\theta}$ under the conditional distribution of $p_\theta(h|v)$, and $\sum_{v,h} p_\theta(v,h)\frac{\partial E_\theta(v,h)}{\partial\theta}$ is the expectation of the energy gradient function $\frac{\partial E_\theta(v,h)}{\partial\theta}$ under the joint distribution of $p_\theta(v,h)$. So, Eq. (9) can be denoted as the following equation:

$$\begin{aligned}\frac{\partial L(\theta)}{\partial \theta} &= -E_{p_\theta(h|v)}\left[\frac{\partial E_\theta(v,h)}{\partial \theta}\right] + E_{p_\theta(v,h)}\left[\frac{\partial E_\theta(v,h)}{\partial \theta}\right] \\ &= -\left\langle \frac{\partial E_\theta(v,h)}{\partial \theta}\right\rangle_{p_\theta(h|v)} + \left\langle \frac{\partial E_\theta(v,h)}{\partial \theta}\right\rangle_{p_\theta(v,h)}\end{aligned} . \qquad (10)$$

The second item in Eq. (9) can be write as below

$$\begin{aligned}\sum_{v,h} p(v,h)\frac{\partial E(v,h)}{\partial \theta} &= \sum_v \sum_h p(v)\, p(h|v)\frac{\partial E(v,h)}{\partial \theta} \\ &= \sum_v p(v) \sum_h p(h|v)\frac{\partial E(v,h)}{\partial \theta} \\ &= \left\langle \sum_h p(h|v)\frac{\partial E(v,h)}{\partial \theta}\right\rangle_{p_\theta(v)}\end{aligned} , \qquad (11)$$

Therefore, we firstly compute $\sum_h p(h|v)\frac{\partial E(v,h)}{\partial \theta}$. We have the following equations:

$$\begin{aligned}\sum_h p(h|v)\frac{\partial E(v,h)}{\partial w_{ij}} &= -\sum_h p(h|v) h_i v_j \\ &= -\sum_{h_i}\sum_{h_{-i}} p(h_i|v)\, p(h_{-i}|v) h_i v_j \\ &= -\sum_{h_i} p(h_i|v) h_i v_j \sum_{h_{-i}} p(h_{-i}|v) \\ &= -\sum_{h_i} p(h_i|v) h_i v_j \\ &= -\left(p(h_i=0|v)\cdot 0\cdot v_j + p(h_i=1|v)\cdot 1\cdot v_j\right) \\ &= -p(h_i=1|v) v_j\end{aligned} , \qquad (12)$$

$$\begin{aligned}\sum_h p(h|v)\frac{\partial E(v,h)}{\partial b_i} &= -\sum_h p(h|v) v_i \\ &= -v_i\end{aligned} , \qquad (13)$$

$$\begin{aligned}\sum_h p(h|v)\frac{\partial E(v,h)}{\partial c_i} &= -\sum_h p(h|v) h_i \\ &= -p(h_i=1|v)\end{aligned} . \qquad (14)$$

Based on the joint probability distribution (Eq. (2)), we can get the following conditional probability:

$$\begin{aligned} p(h_k=1|v) &= p(h_k=1|h_{-k},v) \\ &= \frac{p(h_k=1,h_{-k},v)}{p(h_{-k},v)} \\ &= \frac{p(h_k=1,h_{-k},v)}{p(h_k=1,h_{-k},v)+p(h_k=0,h_{-k},v)} \\ &= \frac{e^{-E_\theta(h_k=1,h_{-k},v)}}{e^{-E_\theta(h_k=1,h_{-k},v)}+e^{-E_\theta(h_k=0,h_{-k},v)}} \\ &= \frac{1}{1+e^{-E_\theta(h_k=0,h_{-k},v)+E_\theta(h_k=1,h_{-k},v)}} \end{aligned} . \tag{15}$$

Due to

$$\begin{aligned} E_\theta(v,h) &= -\sum_{i=1}^{n_v} b_i v_i - \sum_{i=1}^{n_h} c_i h_i - \sum_{j=1}^{n_h}\sum_{i=1}^{n_v} h_j w_{ji} v_i \\ &= -\sum_{i=1}^{n_v} b_i v_i - \sum_{i\neq k}^{n_h} c_i h_i - c_k h_k - \sum_{i=1}^{n_v}\sum_{j\neq k}^{n_h} h_j w_{ji} v_i - \sum_{i=1}^{n_v} h_k w_{ki} v_i \end{aligned} , \tag{16}$$

$$E(h_k=0,h_{-k},v) = -\sum_{i=1}^{n_v} b_i v_i - \sum_{i\neq k}^{n_h} c_i h_i - \sum_{i}^{n_v}\sum_{j\neq k}^{n_h} h_j w_{ji} v_i \ , \tag{17}$$

$$E_\theta(h_k=1,h_{-k},v) = -\sum_{i=1}^{n_v} b_i v_i - \sum_{i\neq k}^{n_h} c_i h_i - c_k - \sum_{i=1}^{n_v}\sum_{j\neq k}^{n_h} h_j w_{ji} v_i - \sum_{i=1}^{n_v} w_{ki} v_i \ , \tag{18}$$

so,

$$-E_\theta(h_k=0,h_{-k},v) + E_\theta(h_k=1,h_{-k},v) = -c_k - \sum_{i=1}^{n_v} w_{ki} v_i \ . \tag{19}$$

Thus, we can get

$$p(h_k=1|v) = \frac{1}{1+e^{-\left(c_k+\sum_{i=1}^{n_v} w_{ki} v_i\right)}} \ . \tag{20}$$

Similarly, we can get the conditional distribution of visible variables

$$p(v_k=1|h) = \frac{1}{1+e^{-\left(b_k+\sum_{j=1}^{n_h} h_j w_{jk}\right)}} \ . \tag{21}$$

At this point, we still need to complete the calculation for Eq. (11). Since the hidden variables cannot be directly observed, we use CD- k algorithm to approximately estimate the edge distribution $p(v)$ through Gibbs sampling in k steps, thus to obtain the solution of $\left\langle \sum_h p(h|v)\frac{\partial E(v,h)}{\partial\theta} \right\rangle_{p_\theta(v)}$. For sample v, we firstly set $v^{(0)} = v$. The details for Gibbs sampling are as follows:

Step 1. Sample the hidden layer through visible layer, i.e., use $p\left(h^{(t-1)} \mid v^{(t-1)}\right)$ to obtain the sample value $h^{(t-1)}$. For $j=1,2,\cdots,n_h$, r_j is randomly generated in the interval [0,1], then the variables in $h^{(t-1)}$ are given by

$$h_j = \begin{cases} 1, if \ r_j < p\left(h_j = 1 \mid v^{(t-1)}\right) \\ 0, otherwise \end{cases}. \tag{22}$$

Step 2. Sample the visible layer through hidden layer, i.e., use $p\left(v^{(t)} \mid h^{(t-1)}\right)$ to obtain the sample value $v^{(t)}$. For $i=1,2,\cdots,n_v$, r_i is randomly generated in the interval [0,1], then the variables in $v^{(t)}$ are given by

$$v_i = \begin{cases} 1, if \ r_i < p\left(v_i = 1 \mid h^{(t)}\right) \\ 0, otherwise \end{cases}. \tag{23}$$

Step 3. Let $t = t+1$. Repeat Step 1 and Step 2 until $t = k$.

We use the $v^{(k)}$ obtained by the above sampling steps to approximately estimate the expectations of $\left\langle \sum_h p(h \mid v) \frac{\partial E(v,h)}{\partial \theta} \right\rangle_{p_\theta(v)}$. Finally, we get the following equations for the gradients of sample v.

$$\frac{\partial L(\theta)}{\partial w_{ij}} = p\left(h_i = 1 \mid v^{(0)}\right) v_j^{(0)} - p\left(h_i = 1 \mid v^{(k)}\right) v_j^{(k)}, \tag{24}$$

$$\frac{\partial L(\theta)}{\partial b_i} = v_i^{(0)} - v_i^{(k)}, \tag{25}$$

$$\frac{\partial L(\theta)}{\partial c_i} = p\left(h_i = 1 \mid v^{(0)}\right) - p(h_i = 1 \mid v^{(k)}). \tag{26}$$

1.2 RBMs for real-valued variables

When RBM is of binary hidden variables and real-valued visible variables, the conditional distribution over the visible variables is usually supposed to be a Gaussian distribution whose mean is a function of the hidden variables[2]. One way of parametrizing Gaussian-Bernoulli RBMs is to use a covariance matrix for the Gaussian distribution. Therefore, we wish to have the following conditional distribution

$$p_\theta(v_i \mid h) = N(\sum_{j=1}^{n_h} h_j w_{ji} + b_i, \sigma_i^2). \tag{27}$$

Based on Eq. (27), the energy function of the RBM with binary hidden variables and real-valued visible variables can be defined as

$$E_\theta(v,h) = \sum_{i=1}^{n_v} \frac{(v_i - b_i)^2}{2\sigma_i^2} - \sum_{j=1}^{n_h} c_j h_j - \sum_{i=1}^{n_v}\sum_{j=1}^{n_h} \frac{v_i}{\sigma_i^2} h_j w_{ji} . \tag{30}$$

While the Gaussian RBM has been the canonical energy model for real-valued data, sampling from $p_\theta(v_i \mid h)$ requires computing the covariance matrix at every iteration of learning. This can be an impractical computational burden for larger visible variables. To simplify the calculation process, we standardize the real-valued data, making the mean of the visible variables v_i is equal to 0 and the variance of that is equal to 1 ($\sigma_i = 1$). In this way, we rewritten the energy function Eq. (30) as

$$E_\theta(v,h) = \sum_{i=1}^{n_v} \frac{(v_i - b_i)^2}{2} - \sum_{j=1}^{n_h} c_j h_j - \sum_{i=1}^{n_v}\sum_{j=1}^{n_h} v_i h_j w_{ji} . \tag{31}$$

For Gaussian RBM, the real-valued units sampled from a Gaussian distribution are seen as the visible units' activation. However, since the gene expression data are very noisy, we discretized the gene expression values into binary values during the Gibbs sampling produce. And we used binary activations instead of the real-valued visible units sampled from a Gaussian distraction which are usually seen as their activations. Because a binary activation contains less information than a real-valued gene expression, using the binary activation to represent a gene expression is helpful to distinguish the genes. This is a very straightforward way to reduce noise in the gene expression data.

According to the above analysis, the parameters learning process for Eq. (31) is similar to that of RBMs with binary variables. The only changes during the parameters learning process caused by the changes of energy function are conditional distributions.

According to the above discuss, we get the following conditional distributions.

$$\begin{aligned} p(h_k = 1 \mid v) &= p(h_k = 1 \mid h_{-k}, v) \\ &= \frac{p(h_k = 1, h_{-k}, v)}{p(h_{-k}, v)} \\ &= \frac{p(h_k = 1, h_{-k}, v)}{p(h_k = 1, h_{-k}, v) + p(h_k = 0, h_{-k}, v)} . \\ &= \frac{e^{-E_\theta(h_k = 1, h_{-k}, v)}}{e^{-E_\theta(h_k = 1, h_{-k}, v)} + e^{-E_\theta(h_k = 0, h_{-k}, v)}} \\ &= \frac{1}{1 + e^{-E_\theta(h_k = 0, h_{-k}, v) + E_\theta(h_k = 1, h_{-k}, v)}} \end{aligned} \tag{32}$$

As

$$\begin{aligned}E_\theta\left(v,h\right)&=\sum_{i=1}^{n_v}\frac{(v_i-b_i)^2}{2}-\sum_{i=1}^{n_h}c_ih_i-\sum_{j=1}^{n_h}\sum_{i=1}^{n_v}h_jw_{ji}v_i\\&=\sum_{i=1}^{n_v}\frac{(v_i-b_i)^2}{2}-\sum_{i\neq k}^{n_h}c_ih_i-c_kh_k-\sum_{i=1}^{n_v}\sum_{j\neq k}^{n_h}h_jw_{ji}v_i-\sum_{i=1}^{n_v}h_kw_{ki}v_i\end{aligned}, \tag{33}$$

$$E\left(h_k=0,h_{-k},v\right)=\sum_{i=1}^{n_v}\frac{(v_i-b_i)^2}{2}-\sum_{i\neq k}^{n_h}c_ih_i-\sum_{i}^{n_v}\sum_{j\neq k}^{n_h}h_jw_{ji}v_i\ , \tag{34}$$

$$E_\theta\left(h_k=1,h_{-k},v\right)=\sum_{i=1}^{n_v}\frac{(v_i-b_i)^2}{2}-\sum_{i\neq k}^{n_h}c_ih_i-c_k-\sum_{i=1}^{n_v}\sum_{j\neq k}^{n_h}h_jw_{ji}v_i-\sum_{i=1}^{n_v}w_{ki}v_i\ . \tag{35}$$

So

$$-E_\theta\left(h_k=0,h_{-k},v\right)+E_\theta\left(h_k=1,h_{-k},v\right)=-c_k-\sum_{i=1}^{n_v}w_{ki}v_i\ . \tag{36}$$

Thus

$$p\left(h_k=1\mid v\right)=\frac{1}{1+e^{-\left(c_k+\sum_{i=1}^{n_v}w_{ki}v_i\right)}}\ . \tag{37}$$

In the same way, we get the conditional distribution of visible variables,

$$\begin{aligned}p\left(v_k=1\mid h\right)&=p\left(v_k=1\mid v_{-k},h\right)\\&=\frac{p\left(v_k=1,v_{-k},h\right)}{p(v_{-k},h)}\\&=\frac{p\left(v_k=1,v_{-k},h\right)}{p\left(v_k=1,v_{-k},h\right)+p\left(v_k=0,v_{-k},h\right)}\\&=\frac{e^{-E_\theta\left(v_k=1,v_{-k},h\right)}}{e^{-E_\theta\left(v_k=1,v_{-k},h\right)}+e^{-E_\theta\left(v_k=0,v_{-k},h\right)}}\\&=\frac{1}{1+e^{-E_\theta\left(v_k=0,v_{-k},h\right)+E_\theta\left(v_k=1,v_{-k},h\right)}}\end{aligned}. \tag{38}$$

As

$$\begin{aligned}E_\theta\left(v,h\right)&=\sum_{i=1}^{n_v}\frac{(v_i-b_i)^2}{2}-\sum_{i=1}^{n_h}c_ih_i-\sum_{j=1}^{n_h}\sum_{i=1}^{n_v}h_jw_{ji}v_i\\&=\sum_{i\neq k}^{n_v}\frac{(v_i-b_i)^2}{2}+\frac{(v_k-b_k)^2}{2}-\sum_{i=1}^{n_h}c_ih_i-\sum_{i\neq k}^{n_v}\sum_{j=1}^{n_h}h_jw_{ji}v_i-\sum_{j=1}^{n_h}h_jw_{jk}v_k\end{aligned}, \tag{39}$$

$$E_\theta\left(v_k=0,v_{-k},h\right)=\sum_{i\neq k}^{n_v}\frac{(v_i-b_i)^2}{2}+\frac{b_k{}^2}{2}-\sum_{i=1}^{n_h}c_ih_i-\sum_{i\neq k}^{n_v}\sum_{j=1}^{n_h}h_jw_{ji}v_i\ , \tag{40}$$

$$E_\theta\left(v_k=1,v_{-k},h\right)=\sum_{i\neq k}^{n_v}\frac{(v_i-b_i)^2}{2}+\frac{(1-b_k)^2}{2}-\sum_{i=1}^{n_h}c_ih_i-\sum_{i\neq k}^{n_v}\sum_{j=1}^{n_h}h_jw_{ji}v_i-\sum_{j=1}^{n_h}h_jw_{jk}\ . \tag{41}$$

So

$$-E_\theta\left(v_k=0,v_{-k},h\right)+E_\theta\left(v_k=1,v_{-k},h\right)=\frac{1}{2}-b_k-\sum_{j=1}^{n_h}h_jw_{jk} \tag{42}$$

Thus

$$p\left(v_k=1|h\right)=\frac{1}{1+e^{-\left(-\frac{1}{2}+b_k+\sum_{j=1}^{n_h}h_jw_{jk}\right)}}. \tag{43}$$

Note that in the learning process of Gaussian-Bernoulli RBM, the sample r_i is generated from a standard normal distribution instead of randomly generated (the Step 2 in CD-k). The rest solving process for Eq. (31) by using gradient up method is same to that of RBMs with binary variables.

[1] Hinton GE: **Training products of experts by minimizing contrastive divergence.** Neural Computation 2014, 14(8):1771-1800.
[2] Hinton GE: **A practical guide to training restricted Boltzmann machines.** Springer Berlin Heidelberg 2012, 9(1):599-619.
